# Quality, then quantity: determinants of autochthony in freshwater food webs

**DOI:** 10.1007/s00442-026-05934-1

**Published:** 2026-07-23

**Authors:** Juliana S. Leal, Angélica L. González, Natália F. Souza, Lúcia F. Sanches, Vinicius F. Farjalla

**Affiliations:** 1 Programa de Pós-Graduação em Ecologia, Universidade Federal do Rio de Janeiro, Rio de Janeiro, RJ Brazil; 2 Department of Biology and Center for Computational and Integrative Biology, The State University of New Jersey, Rutgers, Camden, NJ USA; 3 Departamento de Ecologia, Instituto de Biologia, Universidade Federal do Rio de Janeiro, Rio de Janeiro, RJ Brazil

**Keywords:** Energy pathways, Allochthonous, Stable isotope analysis, Bayesian stable isotope mixing model, Natural microcosms, Tank bromeliads

## Abstract

Understanding how resource quantity and quality shape food webs is a central question in ecology. In freshwater ecosystems, autochthonous organic matter (OM) generally offers higher nutritional quality than allochthonous OM. Although freshwater consumers may preferentially forage on limited but high-quality autochthonous OM, its scarcity often necessitates reliance on more abundant, lower-quality allochthonous OM. Using tank bromeliads as model ecosystems, we conducted a five-month field experiment to test how the dietary contribution of autochthonous OM varies with its quantity. We manipulated incident light to generate a gradient in autochthonous OM availability, evaluating whether its contribution to freshwater consumer diets increased or plateaued due to foraging preferences. We combined Bayesian stable-isotope mixing models with precision-weighted beta regression to quantify diet sources and their drivers. Across the light gradient, autochthonous OM consistently dominated consumer diets (76–95%) despite being > 1,000× less abundant than allochthonous inputs. Median autochthony increased with autochthonous supply, driven by light incidence. Maximum water storage showed a weaker, marginal negative association with autochthony after accounting for light incidence, suggesting that larger tank bromeliad ecosystems exhibited lower autochthony. This pattern may reflect the fact that tank bromeliads are living plants rather than inert containers. Larger seedlings potentially take up more nutrients from the water tanks, which likely reduces nutrient availability to autochthonous primary producers. Overall, our results support a quality-then-quantity hierarchy, in which consumers preferentially consume high-quality autochthonous OM but their realized reliance on this resource is ultimately constrained by the ecosystem’s capacity to produce and retain it.

**Supplementary Information:**

The online version contains supplementary material available at 10.1007/s00442-026-05934-1.

## Introduction

Understanding how resource quantity and quality shape ecological processes is a longstanding theme in freshwater ecology (Tumolo et al. 2023). A central debate in ecology centers on whether the quantity or quality of food resources more strongly determines the structure and functioning of food webs (Brett et al. 2017; Leal et al. 2023). Resource quantity refers to the total amount of food available, whereas quality refers to chemical traits, such as stoichiometry or lability, that influence nutritional value. Classic studies in freshwater food webs emphasize resource quantity as the primary driver of energy flow (e.g., Fisher and Likens 1973; Cummins 1975). Since then, more realistic and holistic approaches have emerged that consider the role of food quality, consumer traits, and cross-ecosystem subsidies in shaping energy transfer pathways (Ings et al. 2009; Thompson et al. 2012; Naman et al. 2022).

This shift in perspective from focusing solely on food quantity to considering food quality has led to a growing emphasis on how different types of resources contribute to energy transfer in freshwater ecosystems (Polis 1991; Nakano and Murakami 2001; Vadeboncoeur et al. 2002). In particular, recent studies have highlighted the contrasting nutritional value of the energy sources in freshwater ecosystems: autochthonous (in situ) and allochthonous (externally derived) organic matter (OM) (Polis et al. 2004; Elser and Hessen 2005; Osakpolor et al. 2023). Autochthonous OM, such as periphyton and phytoplankton, is typically rich in ω-3 polyunsaturated fatty acids and key nutrients, such as nitrogen (N) and phosphorus (P), making it a high-quality resource (Brett and Muller-Navarra 1997; Elser et al. 2000; Sterner and Elser 2002). In contrast, allochthonous OM, such as terrestrial leaf litter, is often nutrient-poor but abundant, sometimes accounting for over 90% of the total OM in some freshwater ecosystems. Because the availability and composition of these resources can vary across environmental conditions, it is essential to understand the environmental drivers that shape their relative contributions to food webs.

One of the most important drivers is light incidence, which can strongly influence the production of autochthonous OM (Maltsev et al. 2023). Photosynthetic activity—and, consequently, the carbon (C) fixation in the autotrophic biomass and the biomass quantity itself— increases almost linearly with rising light levels (Maltsev et al. 2021). However, high light incidence can simultaneously reduce the nutrient content, especially P content of autotrophic biomass, leading to lower nutritional quality (Elser et al. 2003; Solovchenko et al. 2019). This trade-off implies that higher light can increase the total supply of autochthonous OM while decreasing its elemental quality, which increases its C: N and C: P ratios (Wu 2017).

The elemental stoichiometry of food resources (C: N:P) is a well-established indicator of nutritional quality (Danger et al. 2022). When the elemental ratios of available food deviate from consumer requirements, it can limit growth and fitness (González et al. 2014; Lemoine et al. 2014; Danger et al. 2022). However, even high-quality resources may fail to meet consumer demands if they are too scarce (Hill et al. 1992; Andersen et al. 2007; Langerhans et al. 2021). Limited food quantity can impose energetic constraints, while actively searching for rare, high-quality resources increases both energy expenditure and predation risk (Sih 1982; Cruz-Rivera and Hay 2000). As a result, consumers may compensate by relying more heavily on abundant but lower-quality allochthonous OM, even if it is nutritionally suboptimal (Cruz-Rivera and Hay 2000; Flores et al. 2014).

The trade-off between food quality and quantity raises a critical but unresolved question: Is there a threshold level of high-quality food below which consumers shift their behavior toward more abundant but lower quality resources? Understanding such threshold effects is key to unraveling trophic dynamics in allochthonous-dominated ecosystems, where low-quality inputs often dominate (González et al. 2014; Farjalla et al. 2016). Testing this hypothesis requires a system in which both food quality and quantity can be manipulated independently.

Tank bromeliads offer an exceptional model system for this purpose. Like most freshwater ecosystems, they receive high inputs of allochthonous OM from surrounding vegetation. Their rosette-shaped leaves collect rainwater and trap leaf litter, forming discrete, self-contained freshwater ecosystems inhabited by diverse communities of microbes (e.g., bacteria, algae, fungi, protozoa) and aquatic macroinvertebrates (Dézerald et al. 2013, 2014). While allochthonous inputs dominate in most bromeliads (Brouard et al. 2011; Farjalla et al. 2016), these ecosystems can also support autochthonous production via algae that grow on leaf surfaces or in the water column. Light incidence, a key driver of algal growth and stoichiometry, can vary naturally across bromeliads, creating gradients in the quantity and quality of autochthonous OM (Farjalla et al. 2016). This dual-energy pathway, combined with the small and replicable nature of bromeliad ecosystems, provides an excellent experimental framework for disentangling the relative importance of resource quality and quantity in structuring consumer diets (Srivastava et al. 2004; Leal et al. 2022).

This study investigates how the contribution of autochthonous OM to the diet of freshwater consumers is shaped by its quantity and nutritional quality within the ecosystem. To address this, we conducted a five-month field experiment using tank bromeliads as model ecosystems. By manipulating light incidence across 12 levels, we generated a gradient in both the amount and quality of autochthonous OM. Stable isotope analyses were then used to quantify the relative dietary contributions of autochthonous and allochthonous OM to freshwater macroinvertebrates. We tested two competing hypotheses: a quantity-based and a quality-based. The quantity-based hypothesis predicts that consumer reliance on autochthonous OM increases with its abundance due to the energetic cost of locating scarce resources. The quality-based hypothesis predicts that autochthonous OM use is disproportionately high relative to its availability because consumers selectively target high-quality food, even when it is rare.

## Materials and methods

### Study site

**Fig. 1:**
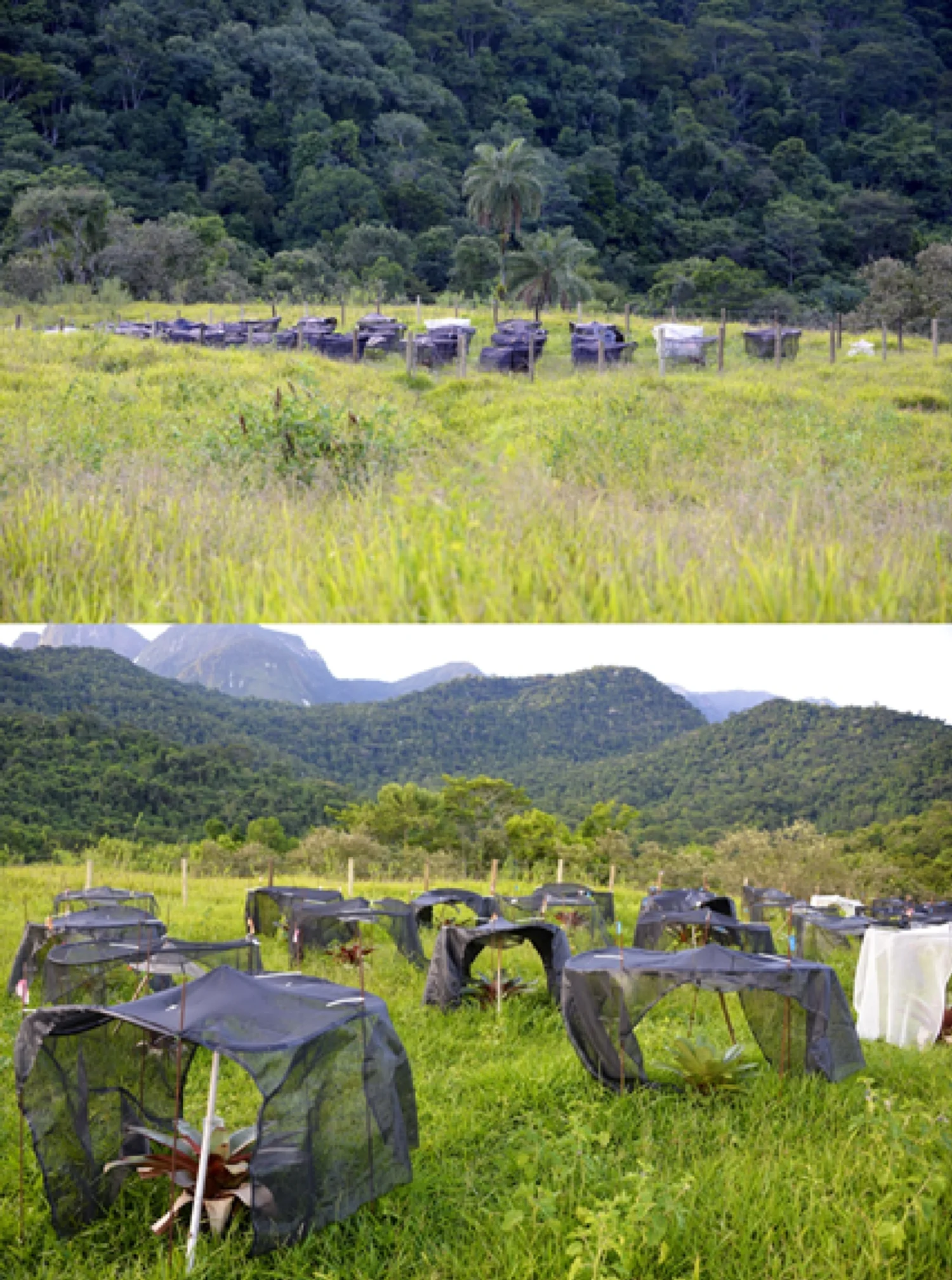
Experiment setup. The experiment was conducted at a site located in a wide-open area adjacent to the rainforest. The details of the light incidence manipulation across the tank bromeliad ecosystems are provided below

The experiment was conducted at the *Reserva Ecológica de Guapiaçu* (REGUA) in Cachoeiras de Macacu (Rio de Janeiro State, Brazil). We selected an open area, surrounded by rainforest but lacking natural canopy cover, to minimize external shading (22°25’34.3” S, 42°45’00.4” W; Fig. 1).

The climate at the site is tropical, with a hot, rainy season from October to March and a dry, mild season from April to September. The mean annual precipitation is 2,600 mm, and the mean annual daily temperature ranges from 14 °C to 37 °C (Kurtz and Araujo 2000). The field experiment was conducted from October 2016 to March 2017, coinciding with the hot, rainy season and the reproductive season for many insect species.

### Experiment design and setup

We conducted a one-factor experiment to manipulate light incidence over tank bromeliads, creating a gradient in autochthonous OM quantity. The experiment consisted of 12 light-incidence levels (i.e., 5.6%, 21.3%, 26.5%, 31.8%, 37.0%, 42.3%, 47.5%, 52.8%, 58.0%, 63.3%, 79.0%, and 100.0%), each replicated 4 times, for a total of 48 experimental units. See the Appendix in the Supplementary Material for details about the canopy cover simulation.

We acquired 48 seedlings of the tank bromeliad *Alcantarea imperialis* (Harms) V. L. Sm. from a licensed nursery and transported them to our field laboratory. This species was selected for its substantial water-volume capacity (mean ± SD: 2.7 ± 0.6 L) and its natural occurrence near the field site (Benzing 2000). At the field laboratory, we thoroughly washed the interior of each bromeliad using a pressure hose and beaker-cleaning brush to remove all residual leaf litter, algae, and associated organisms. After cleaning, each bromeliad was enclosed in a mosquito-netting bag and stored in an open shed for ten days to prevent recolonization.

At the field site, we planted the bromeliads and installed shading shelters above each plant to impose the 12 light levels (Fig. 1). The shelters were constructed by layering various meshes and shade cloth brands to precisely achieve the desired light incidence. A transparent plastic cover beneath the mesh prevented rainwater from entering the bromeliad tanks (Fig. 1). Each bromeliad was randomly assigned to one of the 12 light treatments.

We then added 3.5 g of oven-dried *Eugenia brasiliensis* leaf litter per litter of the bromeliad’s maximum water storage capacity to each tank, simulating allochthonous OM input. This loading matches average natural leaf litter densities in bromeliads (Farjalla et al. 2016). The leaves were freshly fallen, collected from nearby trees, washed, and oven-dried at 40 °C until they reached constant mass. We selected *E. brasiliensis* due to its abundance at the site and its coinciding leaf fall period (Kurtz and Araujo 2000; Donato and Morretes 2007).

To establish autochthonous OM, we inoculated each bromeliad with the laboratory-cultured alga *Ankistrodesmus* sp., by filling tanks to capacity with a solution of 1.5 L of alga diluted in 150 L of spring water (chlorophyll-*a* concentration: 13.33 ± 5.03 µg/L). Laboratory-cultured *Ankistrodesmus* sp. was selected due to its high relative abundance in local bromeliad algal communities (Supplementary Material, Table S1) and to ensure purity of the algal solution. Following the inoculation, the bromeliads were enclosed in mosquito nets for two months to prevent macroinvertebrate colonization. This pre-assembly phase allowed light-driven autochthonous primary production to establish and enabled microbial conditioning of the added leaf litter (Webster and Benfield 1986). After two months, the nets were removed, initiating a three-and-a-half-month natural community assembly within each tank bromeliad.

### Experiment maintenance

Throughout the five-month experiment, bromeliads were watered weekly with spring water to keep their water levels near their maximum capacity. Water volume is an important indicator of hydrological stability in tank bromeliads, supporting the persistence of autochthonous primary producers and reducing the risk of drought stress (Marino et al. 2011). High water levels are also positively associated with greater richness and abundance of aquatic macroinvertebrates (Dézerald et al. 2014). We regularly monitored light incidence at the bromeliad surfaces to ensure our shading shelters maintained the intended light levels (see Supplementary Material, Appendix).

### Sampling and analysis

To estimate the availability of autochthonous OM, we measured chlorophyll-*a* concentrations in bromeliad water biweekly. Water samples (2 mL) were collected from the central tank and four randomly selected lateral tanks. In the laboratory, the chlorophyll-*a* concentration was determined by measuring the fluorescence emission of photosynthetic pigments at 685 nm and excitation at 430 nm (Lorenzen 1966; Suggett et al. 2010), using a Shimadzu RF1501 spectrofluorometer. Background fluorescence was accounted for by filtering an aliquot of the collected water of each bromeliad through GF-1 glass fiber filters (Macherey-Nagel^®^) to create blanks (Carlson and Shapiro 1981). Fluorescence measurements were then converted into chlorophyll-*a* concentration using a calibrated standard curve, allowing us to capture primary productivity dynamics in this turbid, particulate-rich ecosystem.

At the end of the experiment, we sampled both periphyton and aquatic macroinvertebrates. Periphyton was assessed for biomass and nutritional quality as a key source of autochthonous OM. For each bromeliad, we identified the leaf with the most visible periphyton biomass attached to its submerged surface and its opposing leaf. Rectangular Sect.  (2.5 cm x 10 cm) was cut below the water line from both leaves (Supplementary Material, Figure S1). These sections were brushed with a toothbrush and rinsed with 60 mL Milli-Q water. The resulting suspension was homogenized and divided into four subsamples for the following analyses: ash-free dry mass (AFDM), total organic C, chlorophyll-*a*, total N, and total P concentrations.

AFDM was measured after filtration, drying, and ashing. Total organic carbon concentration (TOC) was measured using a TOC-5000 analyzer (Shimadzu^®^). Chlorophyll-*a* was extracted with heated 90% ethanol and measured spectrophotometrically at 665 nm (Nusch and Palme 1975). The remaining subsample was used for total nitrogen and phosphorous analyses. Total nitrogen was determined by the modified Kjeldahl method (Mackereth et al. 1978), and total phosphorus by the molybdenum blue reaction after persulfate oxidation (Golterman et al. 1978).

To estimate autotrophic contribution to periphyton OM biomass, we converted chlorophyll-*a* concentration to algal carbon (hereafter, C_autotrophic_) using a carbon-to-chlorophyll ratio of 27 (Gosselain et al. 2000) and calculated its proportion relative to total carbon (hereafter, C_total_). The remaining fraction of Ctotal was interpreted as non-autotrophic carbon associated with the periphyton matrix (e.g., heterotrophic microbial biomass and fine detrital particles). Thus, we could isolate the effect of light incidence on the carbon fixation by the periphyton photosynthetic activity. The total concentrations of C, N, and P were used to calculate molar C: N, C: P, and N: P ratios, which provided proxies for the nutritional quality of periphytic autochthonous OM (Sterner and Elser 2002; Sanches et al. 2011, 2019).

For stable isotope analysis, we collected visible abundant periphyton from bromeliads. Samples were transported on ice and cleaned of non-algal material under a stereoscope (Leal et al. 2022). This meticulous process ensured accurate isotopic representation of autochthonous OM (Marty and Planas 2008; Yang et al. 2014). We focused on periphytic algae due to their representativeness and feasibility of collection in detritus-rich bromeliad tanks: (i) tank bromeliad ecosystems are turbid and detritus-rich, making particulate organic matter in the water column an unreliable proxy for algal isotopic values (Marty and Planas 2008), (ii) collecting sufficient periphytic algae biomass is more feasible than obtaining free-living algae, and (iii) periphytic and free-living algae often share similar taxonomic compositions, particularly in shallow freshwater ecosystems (Sheath and Hellebust 1978; Moss 1981). In addition, we highlight that the bromeliad water tanks are tiny pools with a few centimeters of depth, which likely strengthens the ecological coupling between free-living and periphytic algae (Schindler and Scheuerell 2002).

Aquatic macroinvertebrate consumers were collected by pipetting water from the tanks, after which the plants were bagged. Both the collected water and bagged bromeliads were transported to the laboratory. In the laboratory, we flushed each bromeliad multiple times, collecting washout in buckets. We recorded bromeliad maximum water storage capacity and the rosette area as habitat size variables (Jocque et al. 2010), potential covariates affecting autochthonous OM availability (Marino et al. 2011). Macroinvertebrates were sorted and identified to Family level, and gut contents were cleared in distilled water for 24 h. Leaf litter was separated, rinsed with distilled water, and set aside.

We analyzed 4 periphyton composite samples, one composite leaf litter sample, and 122 macroinvertebrate samples for δ¹³C and δ¹⁵N. Macroinvertebrate samples were collected for each light-incidence level, with 7 to 14 samples per level (Supplementary Material, Figure S3). All samples were oven-dried at 60 °C for 72 h, ground, and homogenized. We used at least 0.5 mg (macroinvertebrates) and 3 mg (plant and periphyton) dry mass for isotopic analysis. Stable isotope ratios were determined using an NC2500 elemental analyzer interfaced with a Thermo Delta V isotope ratio mass spectrometer at Cornell University.

### Data analysis

To test whether the light manipulation generated a gradient in autochthonous OM availability, we used a generalized least squares (GLS) model. The dependent variable in the model was the mean chlorophyll-*a* concentration, and the independent continuous variables included light incidence, sampling time, and maximum water storage capacity of the tank bromeliads, the latter being known to affect autochthonous OM production (Marino et al. 2011). The GLS model was chosen due to the non-linear relationship between predictors and the response variable, and the presence of temporal autocorrelation in the residuals.

For the GLS analysis, we calculated the mean chlorophyll-*a* concentration (a proxy for autochthonous OM availability) for each light incidence level, averaged across replicates at each sampling time. Multiple models with different variance functions were tested, and the best-fitting model was selected based on the AIC. A constant variance function (VarIdent) was used to account for differences across light incidence levels and sampling times. The optimal model included an interaction between the light incidence and sampling time, along with an Autoregressive Moving Average (ARMA) structure with two autoregressive terms (p) and one moving average term (q), and a cubic spline to address nonlinearity in chlorophyll-*a* dynamics (GLS R^2^ = 0.45).

To evaluate whether light incidence and habitat size affected the autochthonous OM availability, we used linear mixed-effects models (LMM). Dependent variables included periphyton AFDM, chlorophyll-*a* concentration, and C_autotrophic_:C_total_. Independent variables included light incidence, bromeliad maximum water storage capacity, and rosette area, with replicate identity added as a random effect to account for natural variation among bromeliads. All the response variables were log-transformed to meet the LMM assumptions. Similar LMM structures were used to evaluate the effects of light incidence and C_autotrophic_:C_total_ on autochthonous OM quality, considering periphyton C: N, C: P, and N: P ratios as response variables.

To estimate the dietary contributions of autochthonous and allochthonous OM to freshwater consumers, we used a two-isotope (δ^13^C and δ^15^N), two-source (periphyton and leaf litter) Bayesian stable isotope mixing model (SIMM). We pooled the consumers from all four replicates per light treatment to increase statistical precision, acknowledging the sensitivity of SIMMs to sample size. We performed the Bayesian SIMM using the MixSIAR package (version 3.1.12; Stock and Semmens 2016; Stock et al. 2018). In MixSIAR, light incidence was treated as a fixed factor, and the source concentration dependency was incorporated as an informative prior. Trophic enrichment factor values for invertebrates were obtained from the literature (δ^13^C 1.0 ± 0.5‰, δ^15^N 2.5 ± 0.5‰; Caut et al. 2009). We used “extreme” run settings for MCMC sampling in MixSIAR, and assessed convergence with Gelman-Rubin diagnostics in the built-in output routines. Posterior distributions for the proportional dietary contributions of each OM source (see Supplementary Material, Figure S2) were summarized using medians and associated credible intervals.

To test whether consumer autochthony tracked autochthonous OM availability and bromeliad habitat size, we modeled the median autochthonous contribution estimated from the SIMMs using beta regression. We used beta regressions because they are appropriate for modeling fractional outcomes such as the source contribution. Models were fitted using the median autochthonous contribution as the response variable, with candidate predictors including light incidence, bromeliad maximum water storage capacity, and plant rosette area. We also added precision weights to the model structure that reflect the posterior uncertainty in the SIMM estimates of the autochthonous contribution. For each observation, we took its 95% posterior credible interval (from the 2.5th to the 97.5th) and treated half of that interval width as the uncertainty of the observation. Candidate beta regression models were compared based on AICc. Models within ΔAICc ≤ 2 were treated as similarly supported. Among these competitive models, we retained the one that included light incidence and maximum water storage as predictors because it had the highest pseudo-R² (0.50) within the competitive set. Models including rosette area were not retained due to lower AICc support.

All analyses were conducted in R v4.6.0 (R Core Team 2026). GLS models were run using the “gls” function from the nlme package. For the GLMMs, we used the function “lmer” from the lme4 package and assessed the model diagnostics as well as marginal and conditional R² using the performance package. Beta regressions were fitted using the “betareg” function from the betareg package. Candidate beta-regression models were compared using the function “lrtest” from the lmtest package.

## Results

**Fig. 2:**
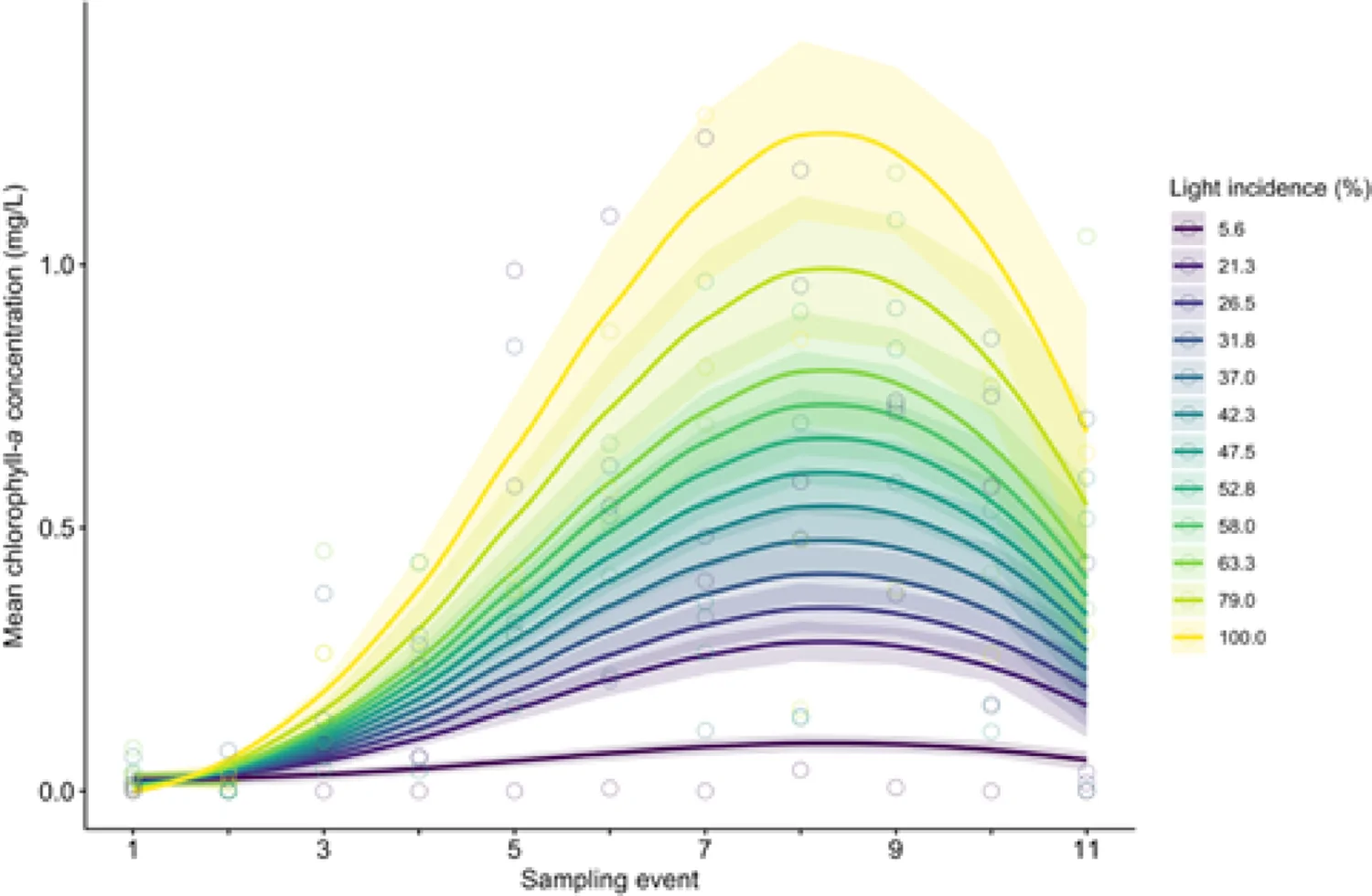
GLS-predicted temporal dynamics of mean chlorophyll-*a* concentration across the light-incidence gradient. Points represent observed mean chlorophyll-*a* concentrations for each sampling event and light-incidence level, and lines represent GLS-predicted temporal trends. Shaded areas indicate the uncertainty around model predictions

Mean chlorophyll-*a* concentration in tank bromeliads varied significantly and non-linearly over time as a function of the interaction between light incidence and sampling time (Table 1). The best-fit GLS model incorporated three time splines to capture temporal fluctuations in this relationship. The interaction between light incidence and the first-time spline had a significant negative effect on chlorophyll-*a* concentration, while the interactions with the second- and third-time splines showed strong positive effects (Table 1). However, the interaction between light incidence and the second time-spline term was stronger than that involving the third time-spline term, indicating that differences among light treatments peaked during the intermediate temporal phase. This pattern is illustrated in Fig. 2, in which it is observable that, at the beginning of the experiment, predicted chlorophyll-*a* concentrations were similar across light-incidence levels. Over time, treatments with higher light incidence showed a stronger increase in chlorophyll-*a* concentration, whereas concentrations declined or stabilized toward the final sampling events.

**Table 1:** Results of the generalized least squares (GLS) model. We calculated the mean chlorophyll-*a* concentration across replicates exposed to the same light incidence level and used it as the response variable in the GLS. The predictors in the model were the level of light incidence and the timing of the sampling event during the experimental period when the chlorophyll-*a* water samples were collected. Due to the non-linear nature of the data, we incorporated three-time splines as interactions with the light incidence variable in the model. Significant results are highlighted in bold

Predictor	Estimate	Std.Error	t-value	*p*-value
Intercept	21.9626	6.6257	3.3148	0.0012
Light incidence	0.2298	0.2217	1.0362	0.3021
**Light incidence: First Time spline**	**-2.4904**	**0.8150**	**-3.0559**	**0.0027**
**Light incidence: Second Time spline**	**24.0886**	**2.4307**	**9.9102**	**< 0.0001**
**Light incidence: Third Time spline**	**6.3658**	**1.3382**	**4.7570**	**< 0.0001**
Correlation Structure: ARMA (2,1)				
Phi1: -0.15 Phi2: 0.13 Theta1: 0.18				

Light incidence had a strong positive effect on periphyton AFDM, chlorophyll-*a* concentration, and C_autotrophic_:C_total_ (Table 2). Among these, periphyton C_autotrophic_:C_total_ was uniquely influenced by both light incidence and bromeliad maximum water storage capacity (Table 2), with habitat size exerting a stronger effect (Estimate = 0.3663, p-value = 0.0248, Table 2). In terms of stoichiometry, C_autotrophic_:C_total_ negatively affected both periphyton C: N and C: P ratios, indicating enhanced nutritional quality with increasing autotrophic biomass (Table 2). However, N:P ratios were not significantly affected. Light incidence had no direct effect on periphyton C: N, C: P, or N: P molar ratios (Table 2).

**Table 2:** Results of the generalized linear models (GLMM) that we used to determine the influence of light incidence and habitat size (i.e., bromeliad maximum water volume and rosette area) on periphyton AFDM (ash-free dry mass), chlorophyll-*a* concentration, and C_autotrophic_:C_total_ as well as the effect of light incidence and the periphyton C_autotrophic_:C_total_ on its C: N, C: P, and N: P molar ratios. Significant results are highlighted in bold. In the model structure, all response variables were log-transformed to meet the GLMM assumptions, and the replicate identity was used as a random factor. The R^2^ of each model is shown in parentheses

Response variable	Predictor	Estimate	Std. Error	df	t-value	*p*-value
AFDM (R^2^ = 0.29)	Intercept	1.3360	0.6084	40.8914	2.1960	0.0338
	**Light incidence**	**0.0094**	**0.0038**	**39.0026**	**2.4690**	**0.0180**
	Maximum water storage	0.0781	0.0980	40.5687	0.7970	0.4303
	Rosette area	-0.1231	0.8521	40.0985	-0.1440	0.8859
Chlorophyll-*a* concentration (R^2^ = 0.32)	Intercept	1.5109	0.6960	40.7642	2.1710	0.0358
	**Light incidence**	**0.0092**	**0.0044**	**40.0685**	**2.1100**	**0.0411**
	Maximum water storage	0.1296	0.1125	41.3856	1.1530	0.2557
	Rosette area	0.1875	0.9460	40.6931	0.1980	0.8439
C_autotrophic_:C_total_ (R^2^ = 0.41)	Intercept	1.1241	0.9700	37.7609	1.1590	0.2537
	**Light incidence**	**0.0126**	**0.0059**	**38.0255**	**2.1260**	**0.0400**
	**Maximum water storage**	**0.3663**	**0.1570**	**39.3452**	**2.3330**	**0.0248**
	Rosette area	-1.0741	1.3348	38.6044	-0.8050	0.4259
C: N (R^2^ = 0.56)	Intercept	4.0342	0.2728	13.9114	14.7890	< 0.0001
	Light incidence	-0.0014	0.0043	39.7516	-0.3310	0.7430
	**C**_**autotrophic**_:**C**_**total**_	**-0.0375**	**0.0060**	**41.9494**	**-6.2760**	**< 0.0001**
C: P (R^2^ = 0.52)	Intercept	6.6229	0.2916	12.9604	22.7160	< 0.0001
	Light incidence	-0.0004	0.0045	38.5984	-0.0970	0.9230
	**C**_**autotrophic**_:**C**_**total**_	**-0.0345**	**0.0061**	**40.8897**	**-5.5990**	**< 0.0001**
N: P (R^2^ = 0.11)	Intercept	2.4959	0.2035	20.3484	12.2630	< 0.0001
	Light incidence	0.0034	0.0035	38.9698	0.9780	0.3340
	C_autotrophic_:C_total_	0.0027	0.0048	40.7988	0.5740	0.5690

Stable isotope values revealed distinct signatures for the OM sources. The mean ± SD δ^13^C value of autochthonous OM was − 27.57 ± 0.84‰ (*n =* 4), while that of allochthonous OM was − 31.41‰ (*n* = 1). Freshwater consumers had a mean ± SD δ^13^C value of -28.64 ± 1.32‰ (*n =* 122). For δ^15^N, autochthonous OM had a mean value of -0.56 ± 2.56‰ (*n =* 4), compared to 2.63 (*n* = 1) for allochthonous OM. Freshwater consumers had a mean ± SD δ^15^N value of 1.41 ± 0.90‰ (*n =* 122) (see Supplementary Material, Figure S3). The consumers were composed of Culicidae (Diptera, 97% of samples), Coenagrionidae (Odonata, 2%), and Corethrellidae (Diptera, 1%).

**Fig. 3:**
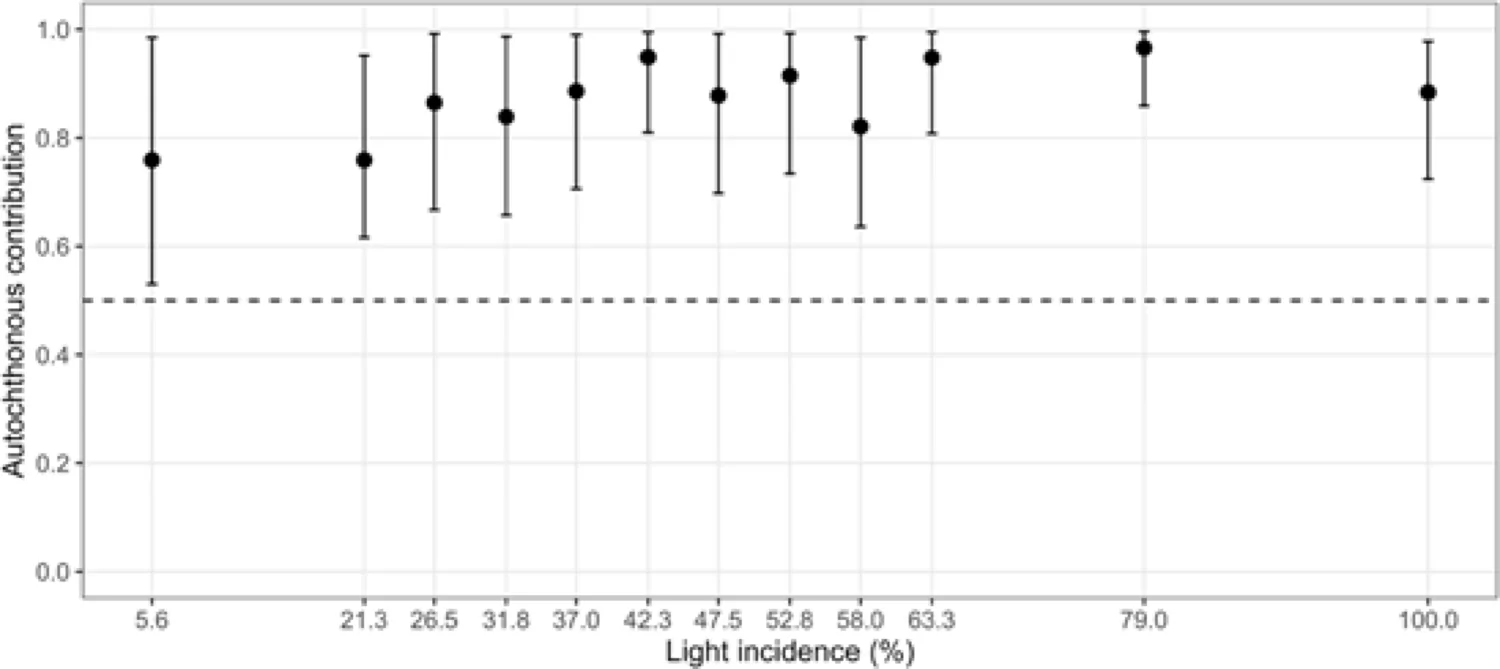
Variation in autochthonous organic matter contribution across light incidence levels. Points represent the median autochthonous contribution to consumers and the error bars its associated 5th and 95th credible intervals, estimated by the Bayesian stable isotope mixing model. The dashed line at 50% contribution highlights that autochthonous OM was the main energy source for freshwater food webs

**Fig. 4:**
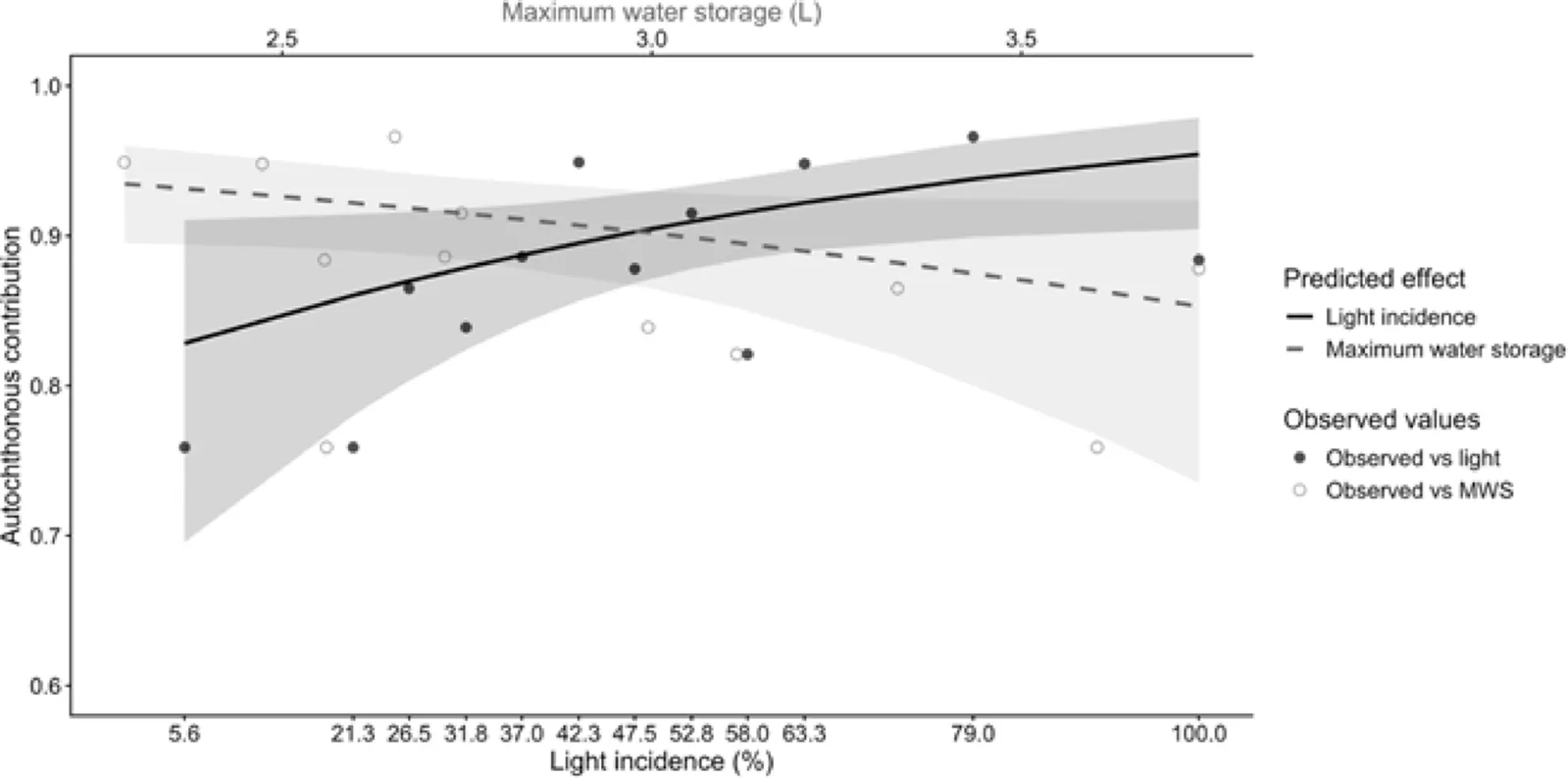
Effects of light incidence and maximum water storage on median autochthonous contribution. Model predictions from the weighted beta regression are shown as lines, with shaded areas representing 95% confidence intervals. Light incidence had a significant positive effect, whereas maximum water storage showed a marginal negative effect. Filled and open circles represent observed values plotted against light incidence and maximum water storage, respectively. MWS is the abbreviation of maximum water storage

Bayesian stable isotope mixing models (SIMMs) showed that autochthonous OM consistently contributed more to freshwater consumer diets than allochthonous OM, regardless of light incidence (Fig. 3). Autochthonous OM contribution ranged from 76% to 95%, while allochthonous contributions ranged from 5% to 24% (Supplementary Material, Table S2). The best-fit beta regression explaining variation in median autochthonous OM contribution identified light incidence as a significant positive predictor of autochthony (Estimate = 0.0155; Std. Error = 0.0075, z = 2.07; p-value = 0.038; Fig. 4), matching the positive trend in Fig. 3. However, maximum water storage had a marginally significant, although considerably strong negative effect on median autochthony (Estimate = -0.6211; Std. Error = 0.3661; z = -1.70; p-value = 0.0898; Fig. 4). In other words, for a given light incidence, larger tank bromeliad ecosystems tended to show lower autochthonous contribution to the diets of freshwater consumers.

## Discussion

In this study, we observed that autochthonous organic matter (OM) was the primary energy source of freshwater food webs, and the degree of autochthony scaled positively with autochthonous OM quantity within the tank bromeliad ecosystem. The influence of autochthonous OM quantity on the degree of autochthony in the food webs was mediated by the light incidence over the tank bromeliads. These results corroborate our quality-based hypothesis, which predicted that the use of autochthonous OM would be disproportionate relative to its availability because consumers selectively target high-quality food. However, they also show that the quantity of autochthonous OM influences the extent to which consumers rely on this food. Together, our findings support a quality-then-quantity hierarchy, in which consumers disproportionately select autochthonous OM, but the realized degree of autochthony scales with its supply.

Classical trophic ecology has emphasized the quantitative question, but there is growing recognition that resource quality constrains energy transfer as strongly as resource quantity (Danger et al. 2022). Optimal foraging theory predicts that both resource quality and quantity shape the feeding strategy of consumers. They can optimize energy gain by favoring the most nutritious food items with low search and handling costs; diets narrow when high-quality items are common and broaden when they are scarce. Here, we showed that this expectation does not necessarily hold. Consistent with many freshwater ecosystems, autochthonous OM quantity was over an order of magnitude lower than that of allochthonous in our tank-bromeliad model ecosystems. Still, autochthonous OM made a dietary contribution exceeding 75% across all incident-light conditions in detritus-rich ecosystems, indicating that resource quality can override scarcity.

Nutritional quality differs markedly between autochthonous and allochthonous organic matter and can strongly constrain the growth and reproduction of primary consumers in freshwater ecosystems(Thomas et al. 2022). When dietary quality is low, consumers assimilate few nutrients per unit ingested, which reduces growth and fecundity (Elser et al. 2012; Sentis et al. 2022). In our system, this growth was likely acute because consumers were insect larvae. About 60% of all freshwater animal species are insects and most of them inhabit fresh waters during their larval stage (Balian et al. 2008; Dijkstra et al. 2014). Consistent with this, multiple studies show that nutritional quality is a major determinant of larval feeding strategies (e.g., Sena et al. 2020; Demi et al. 2022; Cararo et al. 2023). Nevertheless, the role of resource quantity is not negligible.

With light incidence taken as a proxy for autochthonous OM supply, our results indicate that median consumer autochthony tracked the availability of autochthonous OM in the ecosystems. As in typical lentic ecosystems, light incidence is a primary driver of autochthonous OM availability in tank bromeliads (Brouard et al. 2011, 2012). However, autochthonous OM availability is regulated by more complex mechanisms in tank bromeliads, as they are not inert water containers (Takahashi and Mercier 2024). We observed a potential tendency that plants with greater maximum water storage (i.e., larger ecosystems) may show lower autochthony. We argue that although larger tank bromeliads may enhance autochthonous primary production by allowing algal biomass to accumulate over time, they may also represent faster-growing plants with greater nutrient demand. Tank bromeliads take up key nutrients from the water tanks for their growth and reproduction, thereby reducing the availability of autochthonous primary producers (Rogy and Srivastava 2023). Therefore, understanding consumer reliance on autochthonous OM requires examining not only its light-driven supply but also how habitat size influences periphyton biomass, photosynthetic activity, and stoichiometric quality.

Light incidence affected the periphyton quantity-related variable (i.e., AFMD) and photosynthesis-related variables (i.e., chlorophyll-*a* concentration and the C_autotrophic_:C_total_ ratio). Stoichiometric ratios (C: N, C: P, N: P) remained unaffected, indicating that periphyton maintained homeostasis in nutrient uptake despite increased photosynthetic activity. Tank bromeliads are not nutrient-limited freshwater ecosystems due to the decomposition of litter fall and feces in a small water volume (Lopez et al. 2009; Marino et al. 2011), which likely supports this stoichiometric stability. Interestingly, habitat size, measured as maximum water volume capacity, was a stronger driver of the C_autotrophic_:C_total_ ratio than light incidence, corroborating previous findings that habitat volume is key for autotrophic biomass (Marino et al. 2011; Carrias et al. 2025). In its turn, higher autotrophic biomass ratios were associated with lower C: N and C: P ratios, suggesting improved nutritional quality under high-light conditions.

Despite increasing recognition of the importance of food resource quality in the framework of food web energy pathways (Danger et al. 2022), few studies have integrated both quality and quantity experimentally at the ecosystem scale. This study is the first to manipulate light incidence in replicated natural freshwater ecosystems to isolate its effect on autochthony. Still, our findings must be interpreted in light of several limitations. Our ability to use other dietary tracers, such as hydrogen and polyunsaturated fatty acids, was limited by sample mass constraints. We quantified the availability of both free-living algae and periphyton; however, resource quality and stable-isotope composition were assessed only for periphyton due to such sample mass constraints, and the temporal dynamics were tracked with free-living algae alone because periphyton sampling is destructive. Lastly, OM inputs are spatially and seasonally heterogeneous under natural conditions (Doi 2009; Borrelli and Relyea 2022), and autochthonous and allochthonous quantities often covary inversely with canopy cover (e.g., Farjalla et al. 2016). Here, we varied only the autochthonous OM quantity for isolating its effects on the energy pathways of freshwater food webs.

In conclusion, our field experiment revealed that freshwater food webs in tank-bromeliad model ecosystems are predominantly based on autochthonous OM regardless of the light incidence over them, and the fact that it was over an order of magnitude less abundant than allochthonous OM. However, the variation in median autochthony of consumers tracked autochthonous OM supply driven by an incident light production-related effect. These results support a quality-then-quantity hierarchy—consumers preferentially use high-quality autochthonous OM, and the realized reliance on it is set by how much the system can produce and retain. We demonstrated that even in detritus-rich freshwater ecosystems, the higher-quality in situ production can control food-web energetics.

## Supplementary Information

Below is the link to the electronic supplementary material.

Supplementary Material 1

## Data Availability

The data and code used to generate the results presented in this manuscript are available on Zenodo at DOI 10.5281/zenodo.20646700.
