# Supplementary material for: Quality, then quantity: determinants of autochthony in freshwater food webs
Source: Oecologia. 2026 Jul 23;208(8):99. doi: 10.1007/s00442-026-05934-1 (PMC13396072; doi:10.1007/s00442-026-05934-1)
Supplement: Supplementary file 1 — Supplementary Material 1 [file 442_2026_5934_MOESM1_ESM.pdf]

Title: Quality, then quantity: Determinants of autochthony in freshwater food webs

Authors: Juliana S. Leal[1*], Angélica L. González[2], Natália F. Souza[1], Lúcia F. Sanches[1], Vinicius F. Farjalla[1,3]

Affiliations

1 – Programa de Pós-Graduação em Ecologia, Universidade Federal do Rio de Janeiro, Rio de Janeiro, RJ, Brazil.

2 – Department of Biology and Center for Computational and Integrative Biology, Rutgers, The State University of New Jersey, Camden, New Jersey, United States.

3 – Departamento de Ecologia, Instituto de Biologia, Universidade Federal do Rio de Janeiro, Rio de Janeiro, RJ, Brazil.

Corresponding Author: Juliana S. Leal, *leal.julianasilva@gmail.com

Author Contributions

JSL - Conceptualization, Methodology, Data Collection and Analysis, Visualization, and Writing (Original Draft)

ALG - Conceptualization, Methodology, Data Collection and Analysis, Writing (Review and Editing), Funding Acquisition, and Supervision

NFS - Data Analysis, Visualization, and Writing (Original Draft)

LFS - Methodology, Data Collection, and Writing (Review and Editing)

VFF - Conceptualization, Methodology, Data Collection and Analysis, Writing (Review and Editing), Funding Acquisition, and Supervision

Supplementary Material

Table S1. Identification and density of the collected algae taxa in tank bromeliads from the rainforest surrounding the experiment site. We collected a composite water sample from different tank bromeliad species and habitats. The density is given in individual/ L.

Class	Order	Family	Lowest taxonomic level	Density
Bacillariophyceae	Naviculales	Naviculaceae	*Navicula* spp.	1,101
	-	-	Bacillariophyceae sp.1	642
	Thalassiosirales	Stephanodiscaceae	*Cyclotella* spp.	826
Chlorophyceae	Sphaeropleales	Selenastraceae	*Senelastrum* spp.	40,838
			Ankistrodesmus spp.	10,370
		Scenedesmaceae	*Coelastrum* spp.	2,845
Cryptophyceae	Cryptomonadales	Cryptomonadaceae	*Cryptomonas* sp.1	2,478
			Cryptomona sp. 2	1,652
Cyanophyceae	Nostocales	Nostocaceae	*Anabaena grassa*	184
			Anabaena spp.	642
		Aphanizomenonaceae	*Nodularia* spp.	367
	-	-	Cyanophyceae sp.1	826
			Cyanophyceae sp.2	459
			Cyanophyceae sp.3	275
	Synechococcales	Merismopediaceae	*Synechocystis aquatilis*	10,187
Euglenophyceae	Euglenea	Euglenaceae	*Euglena* spp.	1,927
			Euglenophyceae sp.1	275
Trebouxiophyceae	Chlorellales	Oocystaceae	*Oocystis* spp.	1,468
		Chlorellaceae	*Clorella* spp.	4,956
Zygnemaphyceae	Desmidiales	Desmidiaceae	*Cosmarium* spp.	367
		Closteriaceae	*Closterium* spp.	826

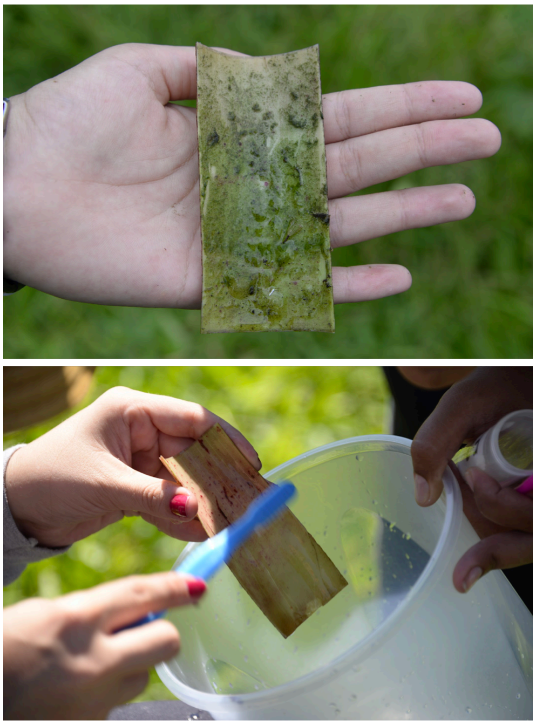

Figure S1. Periphyton sampling in a tank bromeliad leaf at the end of the experiment.

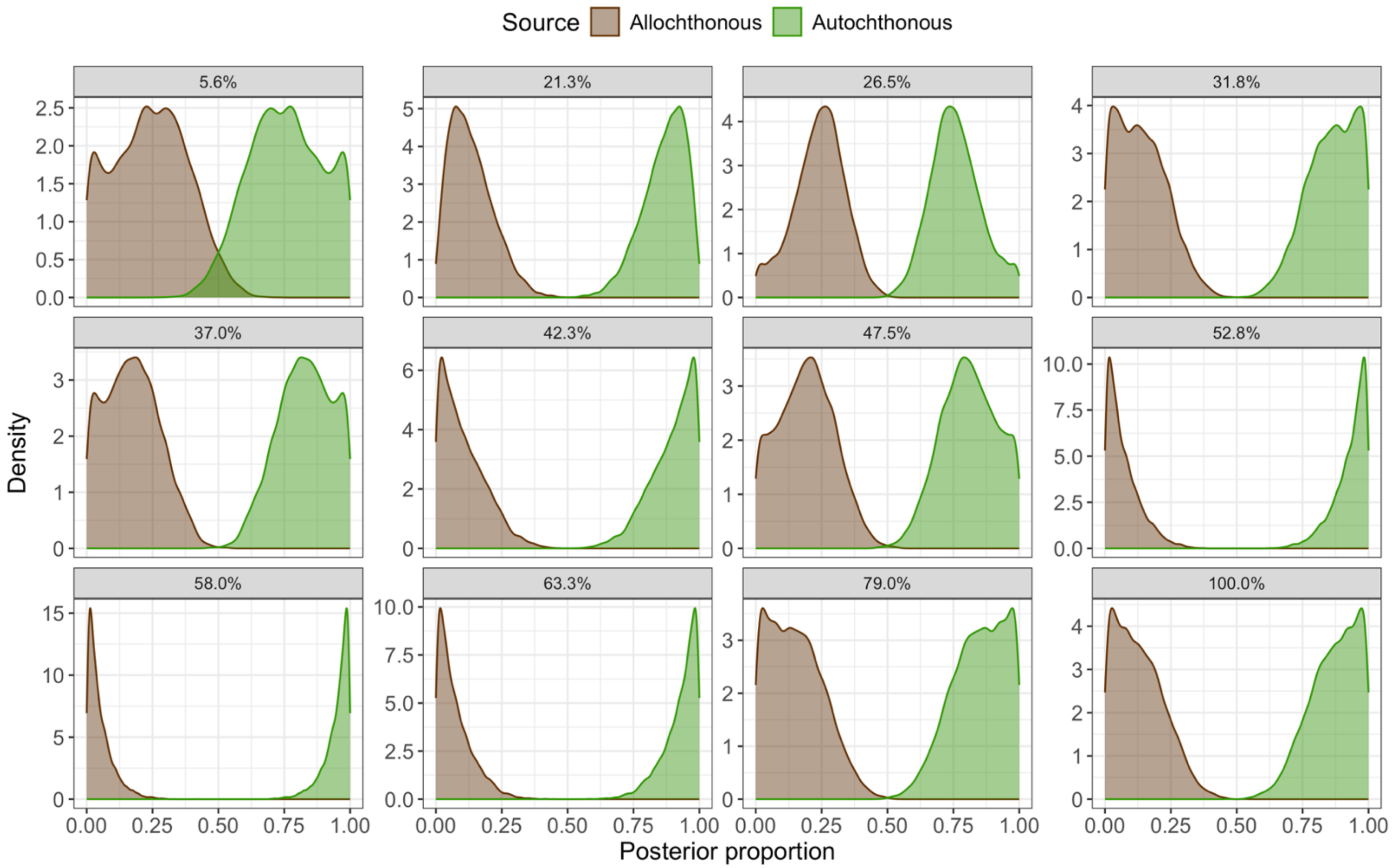

Figure S2. Density plot of the mean dietary contribution of autochthonous and allochthonous resources to the diets of freshwater consumers in each light-incidence level.

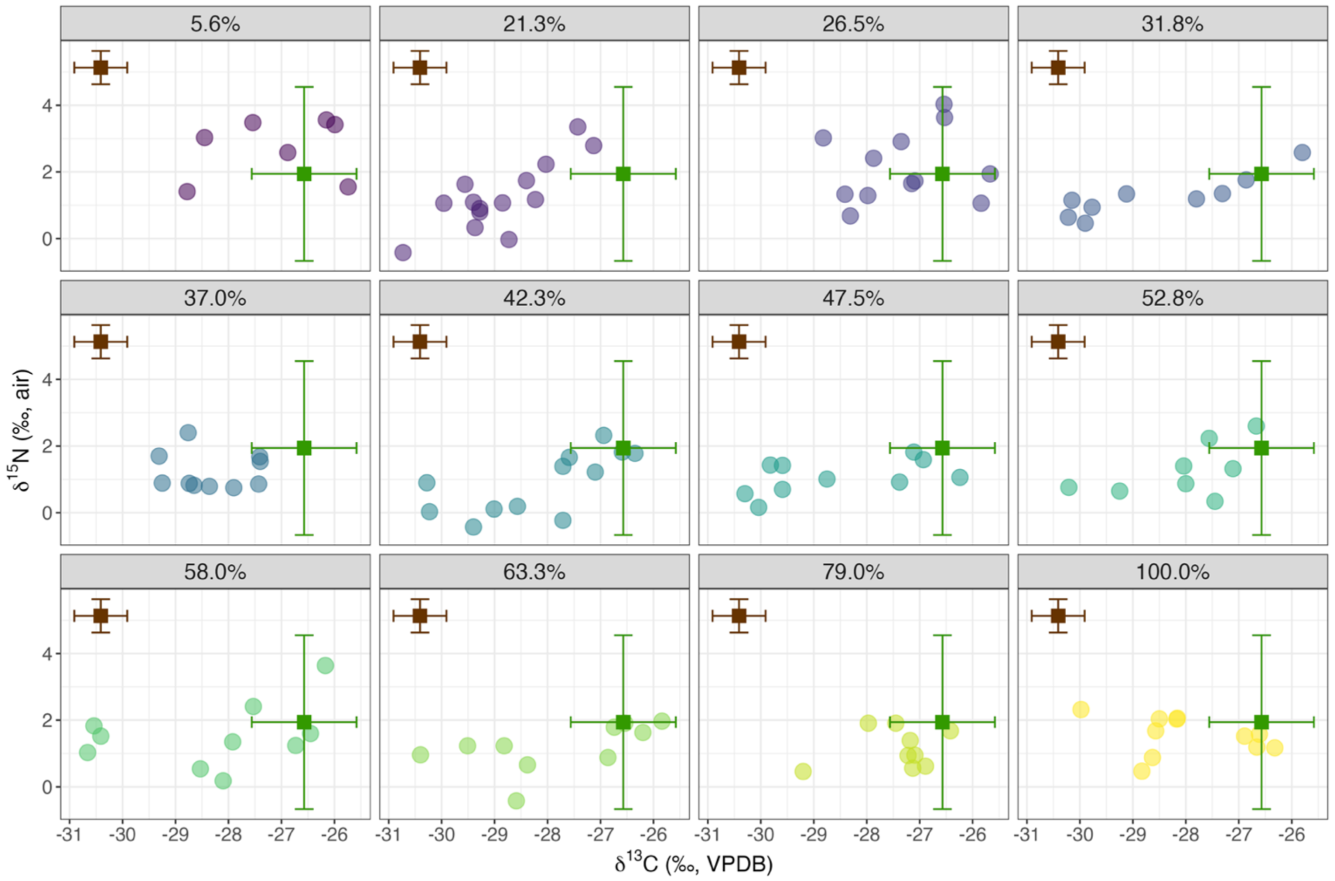

Figure S3. Isospace ($\delta^{13}C \times \delta^{15}N$) of consumers (dots) and sources (squares) in each light-incidence level. Brown and green squares with error bars show TEF-corrected source means ± SD for allochthonous and autochthonous resources, respectively.

Table S2. Estimated mean allochthonous contribution and credible intervals for each light incidence level over the tank bromeliad ecosystems. The sample size of freshwater consumers included in the data analysis is shown in parentheses.

**Light incidence (%)**	**Mean contribution (*n*)**	**Std. deviation**	**Credible intervals**	
			**5th**	**95th**
5.6	0.238 (7)	0.141	0.015	0.470
21.3	0.235 (14)	0.098	0.048	0.384
26.5	0.147 (12)	0.103	0.008	0.333
31.8	0.166 (9)	0.103	0.013	0.343
37.0	0.128 (10)	0.09	0.010	0.294
42.3	0.069 (12)	0.061	0.005	0.190
47.5	0.134 (10)	0.092	0.008	0.302
52.8	0.104 (8)	0.084	0.007	0.266
58.0	0.182 (10)	0.106	0.015	0.364
63.3	0.069 (10)	0.062	0.005	0.192
79.0	0.048 (9)	0.046	0.004	0.141
100.0	0.128 (11)	0.079	0.022	0.276

Appendix

Our light treatments were informed by canopy cover values reported by Farjalla et al. (2016), who found that bromeliad macroinvertebrate diets were predominantly autochthonous under sparse canopy cover (< 25%) and increasingly allochthonous OM under dense canopy cover (> 75%). However, Farjalla et al. (2016) focused on the extremes of light availability and did not explore intermediate levels or quantify autochthonous primary production. To address this gap, we established a continuous gradient of light conditions corresponding to simulated canopy covers of 0%, 20%, 35%, 40%, 45%, 50%, 55%, 60%, 65%, 70%, 75%, and 90%.

To simulate a range of canopy cover levels (0%, 20%, 35%, 40%, 45%, 50%, 55%, 60%, 65%, 70%, 75% and 90%), we used empirical data from Staggemeier & Morellato (2011), who measured canopy openness and photosynthetically active radiation (hereafter " light incidence") in the Atlantic rainforest coastal plain at the Conservation Unit of *Parque Estadual da Ilha do Cardoso*. We converted their canopy openness values into canopy cover percentages using the conversion equation established by Jennings et al. (1999), allowing us to accurately calibrate our light treatments across the gradient.

We measured photosynthetically active radiation (PAR) over the tank bromeliads, at mid-day, on three sunny days in the first, third, and fifth months after the experiment was set up. To do that, we placed a light meter (LI-250A, LI-COR Biosciences) equipped with a US-SQS/LTM spherical micro quantum sensor (Heinz Walz GmbH) immediately above and below the shading shelter. Then, we calculated the proportion of unfiltered light (i.e., the percentage of light incidence reaching the tank bromeliad's surface). To test whether our shading shelters affected the light incidence on the tank bromeliad surface as anticipated, using Spearman's rank correlations between

our expected light incidence levels (i.e., 5.6%, 21.3%, 26.5%, 31.8%, 37.0%, 42.3%, 47.5%, 52.8%, 58.0%, 63.3%, 79.0%, and 100.0%) and the observed levels measured in the field. A Spearman's rank correlation was conducted for each measured three-time point. We ran the Spearman's rank correlations using the "cor.test" function.

We verified that in all our temporal measurements, the expected light incidence levels were highly and significantly correlated with the observed levels of light incidence. In the first temporal measurement we conducted the first month after the experiment setup, the Spearman's rank correlation showed a Spearman's rho = 0.91 and p-value < 0.00001 (S = 1641.3; $n = 48$; Figure A1). In the second temporal measurement we conducted the third month after the experiment setup, the Spearman's rank correlation showed a Spearman's rho = 0.90 and p-value < 0.00001 (S = 1849.9; $n = 48$; Figure A1). In the third temporal measurement we conducted on the fifth month after the experiment setup, the Spearman's rank correlation showed a Spearman's rho = 0.94 and p-value < 0.00001 (S = 1111.5; $n = 48$; Figure A1).

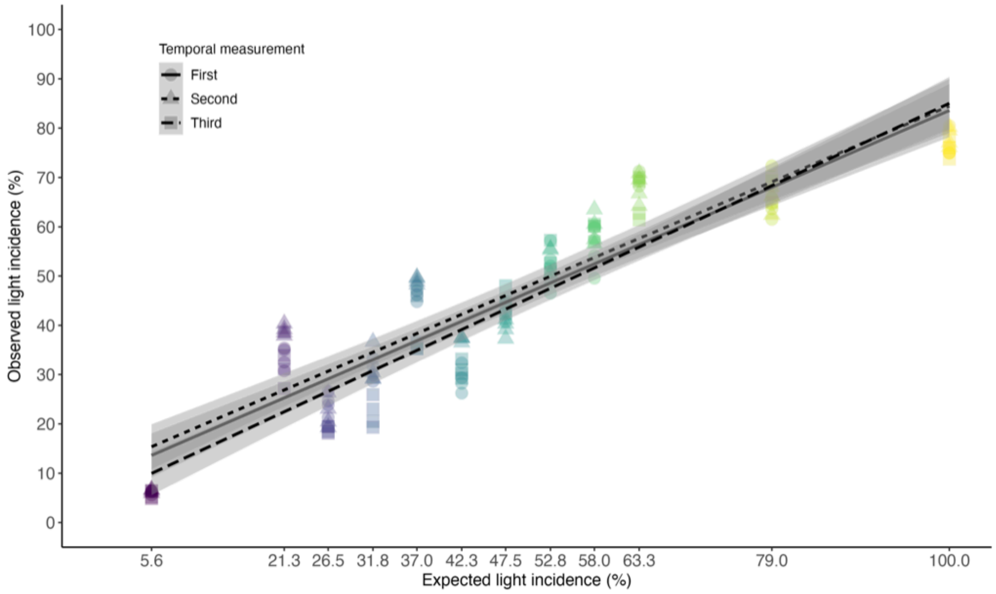

Figure A1. Correlation between the expected and the observed light incidence in each of the three measurements we conducted while we ran the experiment. Gray areas represent the confidence interval.

References

Farjalla VF, Gonzalez AL, Cereghino R, et al (2016) Terrestrial support of aquatic food webs depends on light inputs: a geographically-replicated test using tank bromeliads. Ecology 97:2147–2156. https://doi.org/10.1002/ecy.1432

Jennings SB, Brown ND, Sheil D (1999) Assessing forest canopies and understorey illumination: canopy closure, canopy cover and other measures. Forestry 72:59–74. https://doi.org/10.1093/forestry/72.1.59

Staggemeier VG, Morellato LPC (2011) Reproductive phenology of coastal plain Atlantic forest vegetation: Comparisons from seashore to foothills. Int J Biometeorol 55:843–854. https://doi.org/10.1007/s00484-011-0482-x
